# Tocilizumab improves clinical outcome in patients with active corticosteroid-resistant moderate-to-severe Graves’ orbitopathy: an observational study

**DOI:** 10.3389/fendo.2023.1186105

**Published:** 2023-06-22

**Authors:** Georgios Boutzios, Sofia Chatzi, Andreas V. Goules, Areti Mina, George C. Charonis, Panayiotis G. Vlachoyiannopoulos, Athanasios G. Tzioufas

**Affiliations:** Department of Pathophysiology, National and Kapodistrian University of Athens, Athens, Greece

**Keywords:** Graves orbitopathy, tocilizumab (IL-6 inhibitor), thyroid eye disease, thyroid stimulating antibody, clinical activity score (CAS)

## Abstract

**Background:**

Graves’ orbitopathy (GO) is an autoimmune disorder affecting the orbital fat and muscles. A significant role of IL-6 in the pathogenesis of GO has been described and tocilizumab (TCZ), an IL-6 inhibitor targeting IL-6R has been given in some patients. The aim of our case study was to evaluate the therapeutic outcome of TCZ in non-responders to first line treatments with corticosteroids.

**Methods:**

We conducted an observational study of patients with moderate to severe GO. Twelve patients received TCZ in intravenous infusions at a dose of 8mg/kg every 28 days for 4 months and followed up for additionally 6 weeks. The primary outcome was improvement in CAS by at least 2 points, 6 weeks after the last dose of TCZ. Secondary outcomes included CAS <3 (inactive disease) 6 weeks after TCZ last dose, reduced TSI levels, proptosis reduction by > 2mm and diplopia response.

**Results:**

The primary outcome, was achieved in all patients 6 weeks after treatment course. Furthermore all patients had inactive disease 6 weeks after treatment cessation. Treatment with TCZ reduced significantly median CAS by 3 units (p=0.002), TSI levels by 11.02 IU/L (p=0.006), Hertel score on the right eye by 2.3 mm (p=0.003), Hertel score on the left eye by 1.6 mm (p=0.002), while diplopia persisted in fewer patients (25%) after treatment with TCZ (not statistically significant, p=0.250). After treatment with TCZ, there was a radiological improvement in 75% of patients, while 16.7% showed no response, and in 8.3% of patients deterioration was established.

**Conclusion:**

Tocilizumab appears to be a safe and cost effective therapeutic option for patients with active, corticosteroid-resistant, moderate to severe Graves’ orbitopathy.

## Introduction

Graves’ orbitopathy (GO), also referred to as thyroid eye disease (TED), is an inflammatory autoimmune disorder affecting the orbital fat and muscles that occurs in 30%–50% of patients with Graves’ disease (GD) (1, 2). The disease course varies from inflammation of the extraocular muscles to severe cases of constant diplopia, protrusion, sight threatening with dysthyroid optic neuropathy (DON), and corneal breakdown. Moderate-to-severe orbitopathy affects approximately 5% of patients with GD (2).

According to European Group on Graves’ Orbitopathy (EUGOGO) latest guidelines, first-line treatment of moderate-to-severe GO consists of intravenous corticosteroids (4.5 g cumulative dose of methylprednisolone), either alone or in combination with mycophenolate mofetil; however, the optimal management of corticosteroid-resistant or relapsed cases representing 20%–30% of patients remains a challenge among GO specialists (2, 3).

Higher cumulative doses of corticosteroids, oral corticosteroids combined with steroid-sparing agents such as cyclosporine and azathioprine, surgical orbital decompression, and orbital radiotherapy are recommended as second-line treatments (3). Small RCTs support the use of biologic agents such as rituximab, tocilizumab, and teprotumumab, for GO cases resistant to corticosteroids. After highlighting the significant role of IL-6 in the pathogenetic cascade of TED, tocilizumab (TCZ) has been proposed as an immunomodulator and anti-inflammatory agent that could modify the clinical course of GO (4–6). TCZ is a recombinant humanized monoclonal antibody that blocks the IL-6 receptor and is approved for the treatment of patients with rheumatoid arthritis (RA) and juvenile idiopathic arthritis (JA) (7). Perez-Moreiras et al., in an RCT with 32 patients, demonstrated the efficacy of TCZ on moderate-to-severe corticosteroid-resistant GO disease. Patients received 8 mg/kg TCZ intravenously every 28 days for 4 months over the placebo group, and CAS (Clinical Activity Score) was reduced for at least 2 units, in 93.3% of patients receiving TCZ as opposed to 58.8% of the placebo group. Additionally, improvement according to the EUGOGO classification for severity and exophthalmos were also observed in patients treated with TCZ. Notably, TCZ had no effect on diplopia (8). The aim of our study was to evaluate the therapeutic outcome of TCZ in patients not responding to first-line treatments with corticosteroids.

## Patients

We conducted an observational study of 12 patients with moderate-to-severe GO. The study included patients diagnosed between June 2020 and March 2022. All patients presented with binocular disease and the duration of active GO ranged from 2 to 12 months.

The patients who participated in the treatment protocol were recruited from the autoimmune endocrinopathy outpatient clinic of the Department of Pathophysiology, at Laikon General Hospital, Medical School, National and Kapodistrian University of Athens. All patients had resistant or relapsed disease after receiving first-line treatment with intravenous methylprednisolone (cumulative dose 4. 5g) according to EUGOGO. Corticosteroid relapse was determined as an increase in CAS by at least 2 points, 12 weeks after treatment with corticosteroids. Disease resistance was defined as a non-response to corticosteroids by the end of GC treatment course.

## Methods

Patients received TCZ intravenously at a dose of 8 mg/kg every 28 days for 4 months and were followed up for an additional 6 weeks. The total study duration was 22 weeks. The protocol was approved by the Laikon General Hospital ethical committee after obtaining patients’ informed consent and in compliance with the general data protection regulations (GDPR) of the European Union. All procedures involving human participants were performed in the context of standard of care according to physicians’ judgment and in accordance with the ethical standards of the institutional and/or national research committee as well as with the 1964 Helsinki Declaration and its later amendments.

All patients were evaluated by a single ophthalmologist (GCC) before TCZ and 6 weeks after the last dose. Patients enrolled in the study had moderate-to-severe GO, but some of them during the therapeutic protocol with TCZ developed DON and were treated with either high doses of corticosteroids (1 g of IVMP for three consecutive days) or urgent surgical orbital decompression without cessation of TCZ. We collected and recorded all the relevant medical information including demographic data, medical history of any other systemic or organ-specific autoimmune disease, family history of thyroid disease or autoimmune diseases, date of GD and GO diagnosis, smoking status, and adverse events. Laboratory tests for TSH, T3, FT4, thyroid-stimulating immunoglobulin (TSI), TgAbs, and TPOAbs; complete blood count with differential; biochemistry; thyroid ultrasound; and orbital magnetic resonance imaging (MRI) with orbital muscle size assessment were performed in all patients, before and 6 weeks after the last TCZ dose. All patients enrolled in the study (i) were ≥18 years old at the time of GD diagnosis, (ii) had moderate-to-severe or sight-threatening GO according to EUGOGO classification, and (iii) had active GO with CAS ≥ 3. The seven-point CAS for disease activity according to Mourits was applied in our study (9). Active disease was defined by the presence of three or more of the following symptoms or signs: spontaneous pain, movement-induced pain, eyelid edema, eyelid redness, conjunctival redness, chemosis, and caruncle edema. Severity was evaluated according to EUGOGO classification (3). Moderate-to-severe GO was defined by the presence of two or more of the following features: moderate-to-severe soft tissue involvement, lid retraction ≥2 mm, exophthalmos ≥3 mm above the normal reference range for age and sex, and persistent or recurrent diplopia. Sight-threatening GO included DON or corneal breakdown. Proptosis was measured by a Hertel exophthalmometer and was recorded as Hertel score (10). Diplopia was evaluated both subjectively (based on patient’s perception about the presence or absence of diplopia in primary gaze) and objectively (based on the prism cover test as performed by an ophthalmologist) before TCZ initiation and 6 weeks after the last dose.

Exclusion criteria included mild forms of GO, previous therapy of GO with other biologic agents, pregnancy, active tuberculosis or other active infections, active hepatitis, liver dysfunction (AST or ALT ≥ 1.5 fold the upper limit of normal range), gastrointestinal ulceration, and neutropenia (≤0.5 × 10^9^/lt).

### Medical and surgical treatments performed before TCZ

All patients had been treated with intravenous methylprednisolone (IVMP) pulse therapy, and only 2 (16.7%) received secondary treatment with mycophenolate mofetil at a dose of 1 g/day for 6 months and at least 8 months before TCZ initiation. The time between corticosteroid withdrawal and TCZ infusion was 6 months.

Overall, six patients underwent surgical decompression, five of whom at least 6 months before treatment with TCZ and one during TCZ course. Four patients (4/6) were treated with elective decompression, one patient due to DON before TCZ while one patient developed DON during treatment protocol; the latter received additional course with 1 g of methylprednisolone for three consecutive days with partial response and underwent urgent surgical orbital decompression. However, the response to corticosteroids was not permanent and disease relapsed after treatment cessation; therefore, a second-line treatment with TCZ was considered. All elective decompressions were performed before TCZ treatment in the most severely affected orbit. In five of six patients who underwent decompression, MRIs were performed at two time points (before and after TCZ treatment) and definitely after decompression. In the last patient, MRIs were performed at two time points (before and after TCZ treatment) but after decompression due to DON.

### Assays

The range of normal values was as follows: TSH = 0.27–4.2 μIU/ml, FT4 = 0.7–2.0 ng/dl, and T3 = 0.8–2.0 ng/ml. Thyroid-stimulating immunoglobulins were assessed with Elecsys Anti-TSHR, an electrochemiluminescence method for use in the immunological analyst Cobas e801 (normal values: ≤1.75 IU). TPOAbs were also assessed with a chemiluminometric immunoassay test kit (normal values: <34 IU/ml). The Roche E170 TgAbs is a competitive assay in which patients’ TgAbs competes for binding to solid-phase Tg with ruthenylated human monoclonal TgAbs. The inter-assay precision was 5.4% at 80.8 kU/L and 5.9% at 238.6 kU/L (normal values: <115 IU/ml).

### Outcomes

The primary outcome was improvement in CAS by at least two points, 6 weeks after the last dose of TCZ. Secondary outcomes included CAS <3 (inactive disease) 6 weeks after the last dose of TCZ, improvement in TSI, proptosis reduction by >2 mm, and diplopia response.

### Statistical analysis

Descriptive statistics were used to characterize demographics, clinical information, and outcomes. Continuous variables with normal distribution are presented as mean ± SD, while those without are presented as median (25th, 75th percentile). Normal distribution of continuous variables was assessed with Kolmogorov–Smirnov test. *t*-test and one-way ANOVA (with Bonferroni *post-hoc* correction for multiple comparisons) were used for evaluation of differences in continuous variables with normal distribution, while Mann–Whitney and Kruskal–Wallis were used for continuous variables without. Chi-square tests were used for evaluation of association between nominal variables. Pearson’s and Spearman’s correlation coefficients were used for estimation of correlation between continuous variables. Statistical significance was set at *p* < 0.05. All statistical analyses were conducted using SPSS software v.23.00 (IBM, New York, USA).

## Results

**Table 1** summarizes the demographic and clinical features of patients. Male and female patients were in equal distribution. The mean age ( ± SD) of patients was 58.4 ± 13.4 years old at TCZ initiation. Six and 9 of 12 patients had no family history of GD or other autoimmune disorders, respectively. The majority of them were smokers (66.7%, *N* = 8). In these patients, smoking cessation was recommended.

**Table 1:** Demographic and clinical characteristics of patients with GO (n=12).

**Sex (male/female) (*n*; %)^c^**	6; 50.0%	6; 50.0%
**Age at TCZ initiation (years)^a^**	58.4 ± 13.4	
**Follow-up (years)^b^**	2 (2, 3)	
**Family history (no/yes) (*n*; %)^c^**	6; 50.0%	6; 50.0%
**Other autoimmunities (no/yes) (*n*; %)^c^**	9; 75%	3; 25%
**Anti-TPO (negative/positive) (*n*; %)^c^**	5; 41.7%	7; 58.3%
**Anti-Tg (negative/positive) (*n*; %)^c^**	8; 66.7%	4; 33.3%
**TSH levels (μIU/L)^a^**	0.96 ± 1.03	
**T3 levels (ng/ml)^b^**	1.09 (0.86, 1.42)	
**fT4 levels (ng/dl)^a^**	1.26 ± 0.44	
**Smoking history (no/yes) (*n*; %)^c^**	4; 33.3%	8; 66.7%
**Total thyroidectomy (no/yes) (*n*; %)^c^**	10; 83.7%	2; 16.7%
**Thyroid cancer (no/yes) (*n*; %)^c^**	11; 91.7%	1; 8.3%
**IVMP (no/yes) (*n*; %)^c^**	0; 0%	12; 100%
**IVMP + Secondary (no/yes) (*n*; %)^c^**	10; 83.3%	2; 16.7%
**Ocular surgery (no/yes) (*n*; %)^c^**	6; 50.0%	6; 50.0%

Data are presented as (a) mean ± SD, when the variables are normally distributed; (b) median (25th, 75th percentile), when the variables are not normally distributed; and (c) number of patients and percentage, when the variables are nominal.

TPO, thyroid peroxidase; Tg, thyroglobulin; TSH, thyroid-stimulating hormone; T3, triiodothyronine; fT4, thyroxine.

The primary outcome—at least a two-point reduction of CAS—was achieved in all patients, 6 weeks after the last treatment course. Furthermore, all patients had inactive disease at follow-up, 6 weeks after treatment cessation. One patient developed DON during treatment protocol.

**Figure 1:**
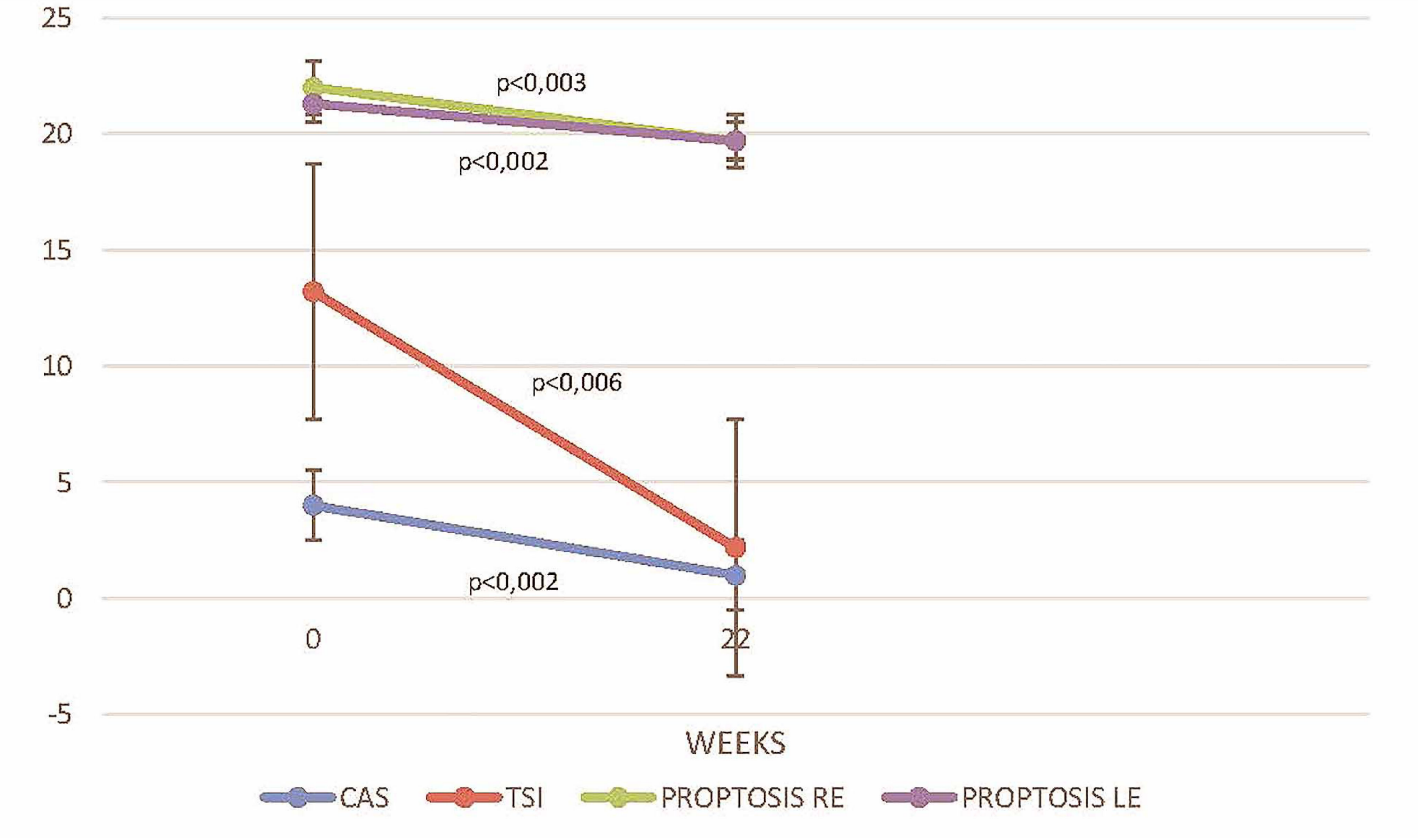
Disease activity assessed with CAS, TSI levels (IU/L), and proptosis for left and right eye (measured in mm) at baseline (week 0) and on follow up 22 weeks after TCZ initiation. RE, right eye; LE, left eye.

**Figure 1** compares critical parameters and scores before and after treatment with TCZ. Treatment with TCZ significantly reduced the median CAS by 3 units (*p* = 0.002), TSI levels by 11.02 IU/L (*p* = 0.006), the Hertel score on the right eye by 2.3 mm (*p* = 0.003), and the Hertel score on the left eye by 1.6 mm (*p* = 0.002). In 4 of 12 (25%) patients who had preexisted diplopia, TCZ had no effect and diplopia remained stable, without significant improvement (*p* = 0.250).

Furthermore, MRI scans revealed that, in the majority of patients, GD affected the superior rectus muscle, the inferior rectus muscle (75%, *N* = 9), and the medial rectus muscle in both eyes (91.7%, *N* = 11); the lateral rectus muscle was also affected in 50% (*N* = 6) of patients in both eyes. After treatment with TCZ, there was an improvement in orbital muscle size in 75% (*N* = 9) of patients, while 16.7% (*N* = 2) showed no response, and in 8.3% (*N* = 1) of patients, there was an increase in orbital muscle size (**Table 2**). These findings are comparable with previous studies (11, 12). Interestingly, the patient who experienced radiological deterioration was not the same patient who developed DON during treatment protocol. Fibrosis or fibro-adipose degeneration was not evaluated with the MRI scans.

**Table 2:** MRI findings: affected muscles at diagnosis and the effect of TCZ treatment.

		No	Right	Left	Bilateral
**MRI-affected muscles**	**Sup. Rectus (*n*; %)**	2; 16.7%	0; 0%	1; 8.3%	9; 75.0%
	**Infer. Rectus (*n*; %)**	1; 8.3%	0; 0%	2; 16.7%	9; 75.0%
	**Med. Rectus (*n*; %)**	1; 8.3%	0; 0%	0; 0%	11; 91.7%
	**Lat. Rectus (*n*; %)**	6; 50.0%	0; 0%	0; 0%	6; 50.0%
	**Sup. Oblique (*n*; %)**	8; 66.7%	0; 0%	0; 0%	4; 33.3%
	**Inf. Oblique (*n*; %)**	9; 75.0%	0; 0%	0; 0%	3; 25.0%
**Effect after TCZ**	**Improvement (*n*; %)**	9; 75.0%			
	**No Response (*n*; %)**	2; 16.7%			
	**Deterioration (*n*; %)**	1; 8.3%			

Data are presented as number of patients and percentage.

TCZ, tocilizumab.

Regarding thyroid function of patients, TSH, T3, and fT4 levels were determined at 0.96 ± 1.03 μIU/L, 1.09 ng/ml, and 1.26 ± 0.44 ng/dl, respectively. Only two patients (16.7%) underwent total thyroidectomy and one patient (8.3%) was diagnosed with thyroid cancer.

Regarding adverse events, four patients experienced an increase in body weight and two patients were diagnosed with hypercholesterolemia, especially an increase in total cholesterol and LDL levels, a finding that appear to be transient, and levels were back to normal within 6 months. No other adverse events were recorded.

Bivariate correlation analysis revealed that T3 levels correlated negatively with the duration of follow-up (CC = −0.791, *p* = 0.04) and positively with CAS after treatment (CC = 0.615, *p* = 0.044); TSI levels before treatment correlated positively with CAS before treatment (CC = 0.594, *p* = 0.42).

## Discussion

Both cellular and humoral immunity are involved in the pathogenesis of GO. Recruitment of T lymphocytes into the orbit induces secretion of a number of cytokines, targeting the orbital fibroblasts, mediating their proliferation and differentiation into mature adipocytes (adipogenesis), and inducing secretion of glycosaminoglycans (GAGs). In the initial active phase of the disease, there is an increased secretion of IL-6, among other pro-inflammatory cytokines (5). B lymphocytes and macrophages play a role in the cascade too, as antigen-presenting and autoantibody-producing cells. In humoral-mediated response, TSH-R expressed on orbital fibroblast membrane forms a complex with IGF-1R. The activation of TSH-R/IGF-1R complexes by TSI further induces adipogenesis and GAG production. IL-6, except from the direct effect on fibroblasts, has shown to increase the expression of TSH-R on orbital fibroblasts and stimulate B cells to produce TSI (4, 6).

Teprotumumab, a monoclonal antibody that inhibits IGF receptor, has been investigated in a phase 3 RCT in patients with moderate-to-severe, active GO, with good response in proptosis, CAS, and diplopia. However, the overall cost of the treatment course is excessive compared to that of TCZ. Moreover, several cases of hearing impairment after treatment with teprotumumab have been reported (13). On the other hand, TCZ, a monoclonal antibody of G1 immunoglobulin inhibiting both the soluble and membrane form of IL-6 receptor, has been proposed as an immunomodulator and anti-inflammatory agent that could modify the disease course of GO (3, 8, 14).

Only few observational studies in the past have shown a beneficial effect of TCZ in GO patients resistant to corticosteroid (15–17). These studies were not randomized and not all patients received IVMP as first-line treatment according to EUGOGO. In the study by Sanchez-Bilbao et al., some patients received conventional immunosuppressive drugs combined with TCZ and therefore the net treatment effect is unclear. In another similar study, TCZ dosage was not standardized in all patients (15). Our study showed a significant improvement of inflammation and proptosis in patients with moderate or severe active GO. In a similar study by Perez-Moreiras et al., the cohort was heterogeneous, as patients were enrolled regardless of the current treatment status, whereas in our study, patients underwent other treatment options at least 6 months before TCZ initiation. In addition, in the same study, non-response to corticosteroids was ill-defined as it was determined after the third intravenous infusion; therefore, some recruited patients had not completed the first-line treatment course of 12 weeks with iv methylprednisolone, which could potentially lead to frank disease remission. In our study, all patients had received methylprednisolone for 12 weeks (cumulative dose 4.5 g), and they were evaluated for second-line treatment with TCZ, after the first-line treatment was completed. Therefore, the improvement observed in CAS, TSI levels, and proptosis could possibly be a result of the synergistic effect of corticosteroids and TCZ and not corticosteroids alone.

The primary outcome of our study, at least two points reduction in CAS, was achieved in all 12 patients, 6 weeks after TCZ discontinuation with significant improvement of inflammation. Congestive symptoms remitted after the second dose of TCZ with constant improvement, until the 6-week follow-up period. Analysis of each component of CAS separately showed significant reduction in all patients. Our results align with those of Perez-Moreiras et al., whereas at week 40, CAS showed improvement by at least two points in 86.7% (*N* = 10) of patients and CAS reached the inactive state of <3 in 80% (*N* = 9) of patients receiving TCZ (8).

Interestingly, regarding the secondary outcomes, improvement in TSI 11.02 IU/L (*p* = 0.006) was established while in those patients who retained high titers, GO remained inactive (18). In a previous study, it was shown that TSI correlated with the disease activity and severity, even after administration of corticosteroid therapy (19). Noteworthily, in the study by Perez-Moreiras et al., the post-treatment TSI levels were not evaluated.

Furthermore, in our study, reduction in proptosis was statistically significant (*p* = 0.002), with 9 of 12 patients meeting the absolute secondary outcome of at least 2 mm in the Hertel score.

Two types of GO have been recognized so far. Type 1 has a slowly progressive course over a long period of time, predominantly affecting the retrobulbar adipose tissue and usually younger, non-smoker patients. In contrast, type 2 evolves rapidly, mainly targets the extraocular muscles, and is observed in older, smoker patients. The latter type leads to more severe disease and is characterized by the occurrence of diplopia (20, 21). TCZ did not seem to significantly improve diplopia in our cohort. This could possibly be explained by the fact that patients with diplopia have diffuse lymphocytic infiltration of extraocular muscles, eventually leading to fibrosis. In addition, although in some patients, muscular inflammation has been partially improved as attested by MRI, the asymmetric muscular involvement and the residual inflammation may explain the persistence of diplopia. Moreover, as mentioned previously, diplopia is observed with higher prevalence in smoker patients (21). Interestingly, two patients showed no response and one patient showed an increase in orbital muscle size in MRI. However, all patients demonstrated improvement in CAS eventually, due to the decrease of the volume of retrobulbar adipose tissue.

Concerning smoking status, the majority of them were also smokers (66.7%, *N* = 8). Smoking is a well-proven independent risk factor for GO. Smokers with GD have a higher prevalence of GO compared to non-smokers; they are at greater risk for severe GO in a dose-dependent way, and the therapeutic outcome after immunomodulatory intervention is poor. The pathophysiological mechanism through which smoking affects GO remains unclear, but it seems that oxygen-free radicals induce orbital hypoxia and stimulate proinflammatory cytokine production, which, in turn, leads to increased adipogenesis (22, 23).

Hyperlipidemia is a known metabolic adverse event in patients with RA and JA treated with TCZ (7). Total cholesterol, high-density lipoprotein (HDL) and triglycerides seem to be significantly elevated with little or no effect in low-density lipoprotein (LDL). This could be due to the blockade of IL-6 receptor and the role of IL-6 in inducing liver apolipoprotein expression (24, 25). Whether TCZ-induced hyperlipemia may adversely affect the course of GO depends largely on the duration of treatment, and therefore, long-term studies are required to draw safe conclusions. One patient developed DON during treatment protocol requiring urgent surgical orbital decompression. CAS remained the same before and after DON, and it was diagnosed after the first dose of TCZs. This patient was older compared to the others; he was a non-smoker and he had a medical history of another systemic autoimmune disease that could potentially affect the treatment outcome. Nevertheless, the patient showed remission regarding the inflammatory component of the GO after TCZ treatment.

Our study has some limitation, with the most important being the small number of patients. Additionally, 6 weeks may be a short follow-up period that did not allow us to evaluate the disease recurrence rate after TCZ cessation. Furthermore, no multivariate LR analysis to identify predictors of TCZ response such as CAS, TSI levels, Hertel score, diplopia, or inflammation of extraocular muscles could be performed, mostly due to the small number of patients included in the study.

Among the strengths of our study is the fact that all patients in our cohort, regardless of their age and disease duration, showed significant clinical response with no serious adverse events. More specifically, eyelid edema, conjunctival hyperemia, chemosis, lacrimation, and proptosis were substantially improved. However, patients did not report remission of diplopia due to the long duration of their orbitopathy while smoking cessation seems to be crucial for treatment outcome.

Treatment of GO has proven to be challenging, especially for patients unresponsive to GCs. The future considerations will be to stratify the benefits and potential risks of this treatment option, and to identify those patients who will respond to TCZ and achieve long-term remission. In conclusion, TCZ appears to be a safe and cost-effective potential therapeutic option for patients with active, corticosteroid-resistant, moderate-to-severe GO.

## Data availability statement

The original contributions presented in the study are included in the article/supplementary material. Further inquiries can be directed to the corresponding author.

## Ethics statement

The studies involving human participants were reviewed and approved by Laikon General Hospital, Athens, Greece. The patients/participants provided their written informed consent to participate in this study.

## Author contributions

GB conceived the idea of the study, designed the study, and wrote the manuscript. SC participated in data collection and wrote the manuscript. AG reviewed and edited the manuscript. AM wrote the manuscript and performed the statistical analysis. GC edited the manuscript. PV reviewed and edited the manuscript. AT reviewed and edited the manuscript and provided revisions to the scientific content of the manuscript. All authors contributed to the article and approved the submitted version.
